# A cross-sectional study describing peripheral neuropathy in patients with symptoms of gastroparesis: associations with etiology, gastrointestinal symptoms, and gastric emptying

**DOI:** 10.1186/s12876-022-02372-0

**Published:** 2022-06-27

**Authors:** Victoria J. Moors, Kathleen D. Graveran, Dariush Shahsavari, Henry P. Parkman

**Affiliations:** 1 Lewis Katz School of Medicine at, Temple University, 3500 North Broad Street, Philadelphia, PA 19140 USA; 2 Temple University Hospital, 3401 North Broad Street, Philadelphia, PA 19140 USA

**Keywords:** Idiopathic gastroparesis, Peripheral neuropathy, NTSS-6, PAGI-SYM

## Abstract

**Background:**

Peripheral neuropathy (PN) is present in diabetic gastroparesis but is not described in idiopathic gastroparesis.

**Aims:**

(1) Determine prevalence of PN in idiopathic gastroparesis; (2) assess if patients with symptoms of gastroparesis and PN differ in gastric emptying and symptoms, both gastrointestinal and psychosocial, from patients without PN (nPN); (3) compare this relationship to that in other etiological groups.

**Methods:**

250 patients with symptoms of gastroparesis underwent gastric emptying scintigraphy and answered questionnaires including severity of gastroparesis symptoms using the Gastroparesis Cardinal Symptom Index (GCSI) and presence of peripheral neuropathy using the Neuropathy Total Symptom Score-6 (NTSS-6).

**Results:**

PN, defined by NTSS-6 > 6, was present in 70/250 (28%) patients: 22/148 (15%) idiopathic, 33/61 (54%) diabetic, and 11/32 (34%) postsurgical (*p* < 0.01). Among 148 patients with symptoms of idiopathic gastroparesis, defined as non-diabetic, non-postsurgical, and not caused by a known disorder such as Parkinson’s or connective tissue disease, symptoms of gastroparesis were more severe in PN than nPN: bloating (4.05 ± 1.17 vs. 2.99 ± 1.61, *p* < 0.01), abdominal distension (3.86 ± 1.49 vs. 2.45 ± 1.68, *p* < 0.01), and upper abdominal pain (3.64 ± 1.22 vs. 2.71 ± 1.78, *p* = 0.03). Ninety-nine idiopathic patients underwent gastric emptying scintigraphy: 7/43 (16%) patients with delayed gastric emptying and 9/56 (16%) patients with normal gastric emptying had PN. Among patients with idiopathic gastroparesis, abdominal distension (4.43 ± 0.53 vs. 2.89 ± 1.68, *p* = 0.01) was more severe in PN than nPN. The association of PN and worse gastrointestinal symptoms was not as apparent in patients with symptoms of diabetic or postsurgical gastroparesis.

**Conclusions:**

PN was present in 70/250 (28%) of patients with symptoms of gastroparesis and was present to a lesser extent in idiopathic than diabetic gastroparesis. The presence of PN in IG was associated with more severe gastroparetic symptoms than in nPN. Screening for PN may help identify a gastroparesis cohort with peripheral neuropathy who are more symptomatic.

## Background

Gastroparesis describes a gastric motility disorder characterized by delayed gastric emptying (GE) with symptoms of nausea, vomiting, early satiety, and postprandial fullness [1]. Symptoms are not well correlated with GE and some patients can have symptoms of gastroparesis with normal GE [2, 3]. There are three primary etiologies: diabetic gastroparesis (DG), postsurgical gastroparesis (PSG), and idiopathic gastroparesis (IG). DG is associated with autonomic dysfunction, vagal dysfunction, and peripheral neuropathy (PN). IG is associated with autonomic dysfunction, but not vagal dysfunction [4, 5]. The strong association between diabetes and PN is well-known, but the presence of PN in IG is not described.

Symptoms of PN vary based on which sensory, motor, and autonomic fibers are affected. Large-diameter sensory fibers relay vibratory sensation and proprioception while small-diameter fibers transmit pain and temperature sensation. Sensory symptoms, which usually present before autonomic symptoms, can include a loss of sensation of vibration, touch, and/or pain. Sensory and motor neurological tests can be performed to assess the extent of peripheral nerve damage, such as the Romberg test for proprioception and pin prick tests for sensation. Nerve conduction studies and electromyography can help determine if large-fiber PN is present but may be normal in small-fiber PN [6]. Autonomic dysfunction originates from damage to the small-fibers [7]. Several questionnaires have been used to help diagnose PN, but they have traditionally been time-consuming and difficult to interpret [8]. The Neuropathy Total Symptom Score-6 (NTSS-6) is a shortened six question questionnaire that has been developed and validated for diabetic peripheral neuropathy [8]. Using this questionnaire, the NIH Gastroparesis Consortium found peripheral neuropathy to be present in 45% of patients with DG [9]. Therapies for diabetic PN are used for abdominal pain in gastroparesis, which can be difficult to treat. Patients with IG often have more severe abdominal pain than patients with symptoms of DG [1]. Elucidation of PN in IG may serve to better understand the pathophysiology and symptoms of IG, and possibly assist in deciding treatments.

Since autonomic dysfunction is present in IG, and autonomic dysfunction is a subtype of PN, we hypothesized that peripheral neuropathy may also be present in IG. The aims of this study were to: (1) Determine prevalence of PN in IG; (2) assess if patients with symptoms of gastroparesis and PN differ in GE and symptoms, both gastrointestinal and psychosocial, from patients without PN (nPN); (3) compare this relationship to that in other etiological groups.

## Methods

### Patient enrollment and classification

**Fig. 1:**
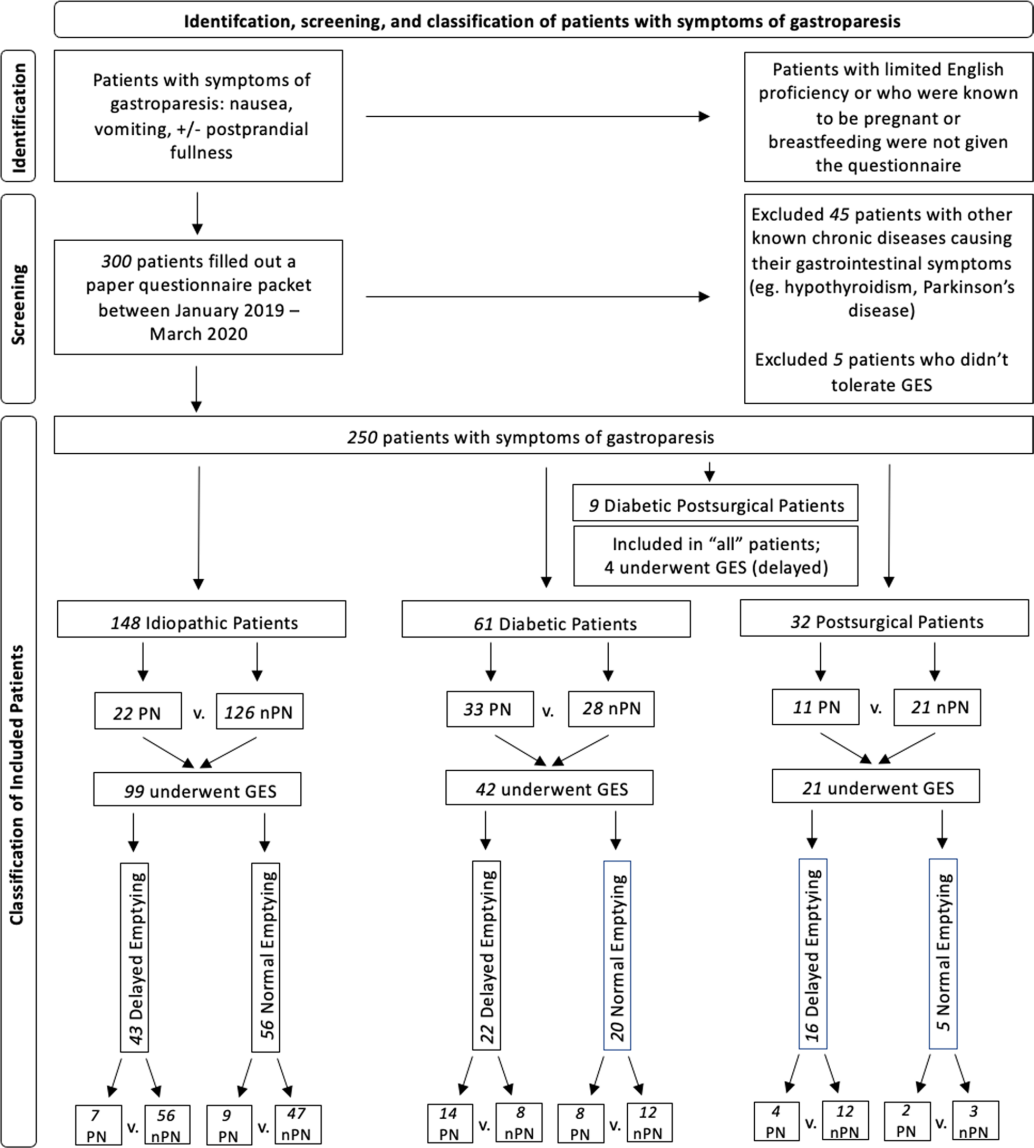
Patient enrollment and classification

Figure 1 follows a PRISMA model and outlines how patients with symptoms of gastroparesis (nausea, vomiting, and postprandial fullness) were organized [10]. These patients were evaluated in the Gastroenterology Section of our tertiary care center between January 2019 and March 2020 and filled out questionnaires detailing symptoms and demographic data. The following patients were excluded: patients known to be pregnant or breastfeeding, patients with limited English proficiency, patients who could not tolerate gastric emptying scintigraphy (GES), and patients with other known chronic diseases causing their gastrointestinal symptoms, such as hypothyroidism.

We evaluated patients with symptoms of gastroparesis. Gastroparesis includes the cardinal symptoms of nausea, vomiting, early satiety, and postprandial fullness in addition to delayed GE [3]. In this study, some patients had normal emptying. “Patients with symptoms of gastroparesis” includes patients with normal and delayed GE and patients without GE data. IG, DG, PSG, and diabetic postsurgical (DPSG) refer to patients with symptoms of gastroparesis and delayed GE as assessed by GES.

### Study procedures

Presence of PN was determined by the NTSS-6, a questionnaire developed for diabetic PN but used for all patients in this study, including non-diabetic. Scoring is based on a combined value of frequency and severity for a total subscore: 1.00 corresponds to “mild and occasional (less than 1/3 of the time),” 1.33 corresponds to “mild and often (1/3 to 2/3 of the time),” 1.66 corresponds to “mild and almost continuous (more than 2/3 of the time).” The increment in thirds repeats with “moderate” symptoms at the 2.00, 2.33, 2.66 levels and “severe” symptoms at the 3.00, 3.33, 3.66 levels. The original Bastyr article on NTSS-6 defines “mild” to mean that the symptoms do not interfere with daily living and require no treatment, “moderate” to mean that the symptoms interfere with or restrict at least one aspect of daily living or treatment is required, and “severe” to mean that the symptoms interfere with daily living even with treatment [8]. Scores > 6 out of a maximum score of 21.96 indicate the presence of PN with increased scores correlate with increased severity of and frequency of the six main symptoms of peripheral neuropathy: pain, burning, “prickling” or “tingling,” numbness without “prickling,” stabbing pain, and sensitivity [8].

The severity of gastroparesis symptoms was assessed by the Patient Assessment of Gastrointestinal Disorders-Symptoms Severity Index (PAGI-SYM) which includes the Gastroparesis Cardinal Symptom Index (GCSI) [11, 12]. Each symptom is graded by the patient on the severity over the last two weeks from zero to five: 0 = none, 1 = very mild, 2 = mild, 3 = moderate, 4 = severe, and 5 = very severe. The PAGI-SYM includes 6 subscales and is an average score of each subscale’s average score: nausea/vomiting, postprandial fullness/early satiety, bloating, upper abdominal pain/discomfort, lower abdominal pain/discomfort, and heartburn/regurgitation [13]. The GCSI scores the averages of the first three subscales.

Additional questionnaires included a history of co-morbidities, history of the patient’s experience with gastroparesis including eating habits, treatments, and medications, and the Hospital Anxiety and Depression Score (HADS). For the HADS, scores are tallied based on anxiety or depression related questions. A score of 0–7 is “normal,” 8–10 is “borderline abnormal,” and 11–21 is “abnormal” [14].

Patients underwent GES if it had not been performed in the recent past (defined as within the past twelve months of filling out the questionnaire). GES was performed over 4 h using the standard low-fat, Eggbeaters® meal to measure solid emptying [15–17]. Patients were instructed to stop medications that could affect GI motility (e.g., prokinetics and opioid analgesics) for 72 h prior to the study and to come to the Nuclear Medicine Section in the morning after fasting overnight. Diabetic patients were instructed to take only their long-acting insulin in the morning prior to coming in, and then were given their short acting insulin or oral hypoglycemic medication with the test meal. Diabetic patients had glucose levels checked to ensure < 270 mg/dL, with appropriate treatment measures taken if hyperglycemia (> 270 mg/dL) was detected. The meal consisted of the equivalent of two large eggs radiolabeled with Tc-99m sulfur colloid served with two pieces of white bread and jelly. This was served with 120 mL water. Following meal ingestion, imaging was performed at 0, 0.5, 1, 2, 3 and 4 h with patient upright for measuring GE of Tc-labeled solids. Gastric retention was analyzed as the percent of radioactivity retained in the stomach over time using the geometric center of the decay-corrected anterior and posterior counts for each time point. Gastric retention of Tc-99 m > 60% at 2 h and/or > 10% at 4 h was considered evidence of delayed GE of solids [15].

### Statistical analysis

For this cross-sectional analysis, data from questionnaires and GES were entered into a Microsoft Excel® spreadsheet. Patients were categorized by etiology: [1] idiopathic (cause not due to diabetes, post-gastric surgery, or other known chronic disease); [2] diabetic (either Type I or Type II); [3] diabetic postsurgical; and [4] non-diabetic postsurgical (not diabetic and surgery limited to stomach or esophagus but excluded the gallbladder and non-abdominal surgeries). Within each etiological group, patients were further categorized into and compared by the presence of peripheral neuropathy (PN) or no peripheral neuropathy (nPN). In addition, patients with symptoms of gastroparesis and delayed GE were further compared exclusive of patients with symptoms of gastroparesis and normal GE.

Data is either presented as mean ± standard deviation or number (%). Two-tailed *t* tests and one-way ANOVA or Mann–Whitney and Kruskal–Wallis tests were used to compare groups depending on data normality. Correlation of gastric retention to PN severity was tested using Pearson’s Correlation. Comparison of percentage values, such as sex distribution and neuromodulator use, were analyzed using Chi Square tests. In this exploratory study, no statistical adjustments for multiple comparisons were made. Statistical analysis was performed using Excel and the Real Statistics Resource Pack software (Release 7.6). Copyright (2013–2021) Charles Zaiontz. www.real-statistics.com [18].

## Results

### Prevalence of PN and delayed GE

**Fig. 2:**
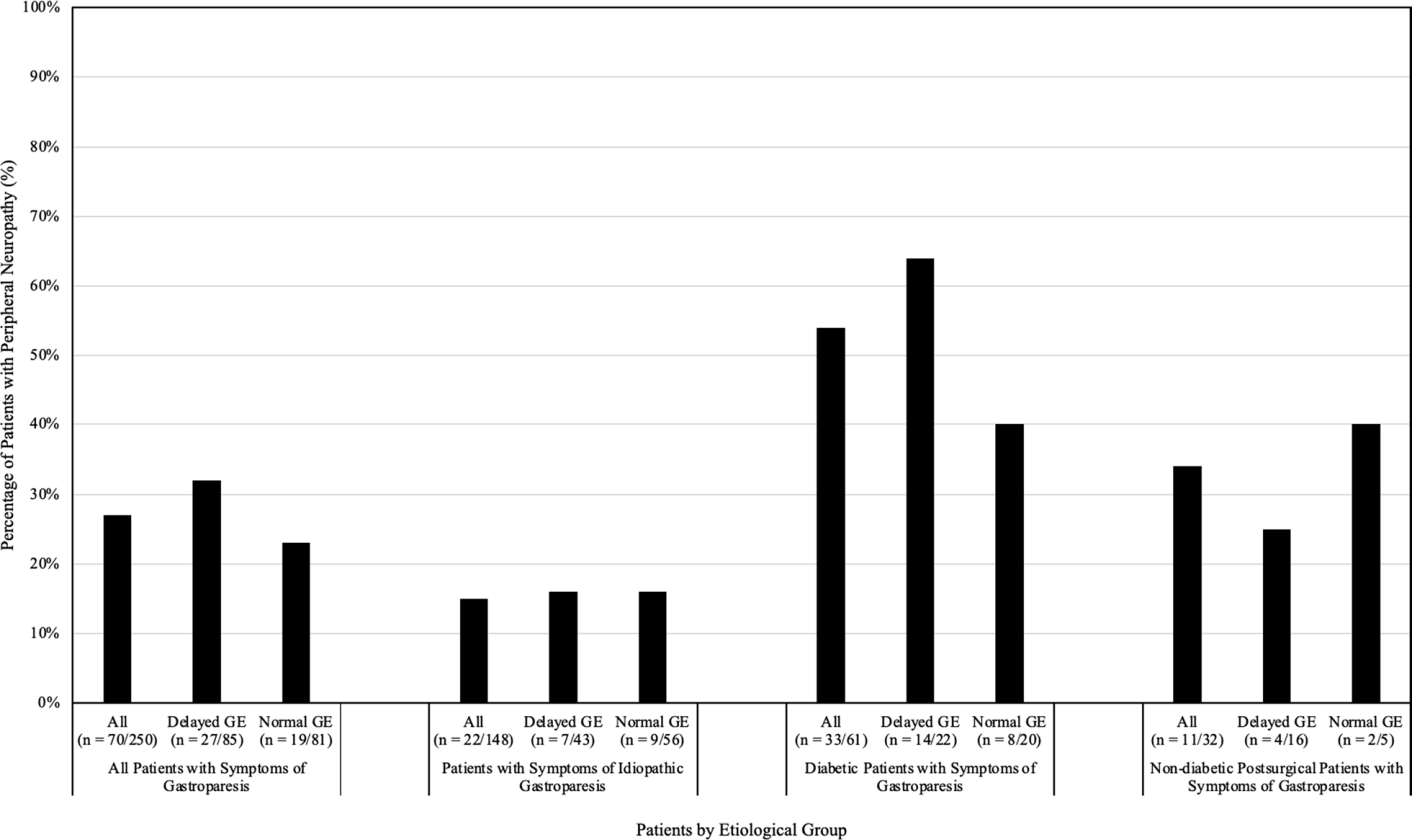
Prevalence of peripheral neuropathy, as determined by the NTSS-6, among patients with symptoms of gastroparesis

250 patients with symptoms of gastroparesis were assessed; Fig. 2 displays the prevalence of PN within each etiological group.

PN as determined by NTSS-6 > 6 was present in 70/250 (28%) patients; 166 patients underwent GES. PN was present in 27/85 (32%) patients with delayed GE and 19/81 (23%) patients with normal GE (*p* = 0.490). Among patients with symptoms of idiopathic gastroparesis, PN was present in 22/148 (15%), 7/43 (16%) patients with delayed GE, and 9/56 (16%) patients with normal GE (*p* = 0.962). PN was present in 33/61 (54%) diabetic patients with symptoms of gastroparesis, 14/22 (64%) patients with delayed GE, and 8/20 (40%) patients with normal GE (*p* = 0.304). Among non-diabetic postsurgical patients with symptoms of gastroparesis, PN was present in 11/32 (34%), 4/16 (25%) patients witih delayed GE, and 2/5 (40%) patients with normal GE (*p* = 0.741). When comparing the prevalence of PN among each etiology, inclusive of patients who hadn’t undergone GES, the *p* value was < 0.005.

Table 1 describes clinical characteristics of patients with symptoms of gastroparesis. Those with PN were older than patients with nPN (46 ± 16 vs. 42 ± 17, *p* = 0.048), but both groups reported a similar duration of gastroparesis symptoms (10 ± 15 years vs. 6 ± 9, *p* = 0.238). The 7/70 (10%) patients with PN reported slightly more neuromodulator use than 6/180 (3%) nPN (*p* = 0.033); neuromodulators include gabalin, pregabalin, mirtazapine, and tricyclic antidepressants (nortriptyline and amitriptyline). Otherwise, patients' age, sex, and duration of symptoms were similar between PN and nPN cohorts within each etiological group.

**Table 1:** Clinical characteristics of patients with symptoms of gastroparesis

	All^a^			Idiopathic			Diabetic			Postsurgical		
	(n = 250)			(n = 148)			(n = 61)			(n = 32)		
	PN	nPN	*p* value	PN	nPN	*p* value	PN	nPN	*p* value	PN	nPN	*p* value
	n = 70	n = 180		n = 22	n = 126		n = 33	n = 28		n = 11	n = 21	
Prevalence within specific etiological group	28%	72%	< 0.005*	15%	85%	< 0.005*	54%	46%	< 0.005*	34%	66%	0.390
Age (years)	46 ± 16	42 ± 17	0.048*	38 ± 17	40 ± 17	0.814	48 ± 13	40 ± 15	0.056	55 ± 14	53 ± 14	0.736
Duration of Gastroparesis (years)	10 ± 15	6 ± 9	0.238	10 ± 11	6 ± 10	0.585	7 ± 11	6 ± 9	0.726	6 ± 9	10 ± 12	0.575
n females (%)	51 (73%)	144 (80%)	0.221	18 (82%)	110 (87%)	0.561	22 (67%)	19 (68%)	0.921	8 (73%)	13 (62%)	0.54
BMI (kg/m^2^)	29 ± 8	26 ± 7	0.009*	27 ± 7	25 ± 7	0.291	31 ± 8	29 ± 6	0.278	27 ± 12	25 ± 5	0.796
n who report neuromodulator use (%)	7 (10%)	6 (3%)	0.033*	2 (9%)	5 (4%)	0.296	4 (12%)	1 (4%)	0.225	1 (9%)	0 (0%)	0.160

n who underwent GES	All PN n = 46	All nPN n = 120	*p* value	Idiopathic PN n = 16	Idiopathic nPN n = 83	*p* value	Diabetic PN n = 22	Diabetic nPN n = 20	*p* value	Postsurgical PN n = 6	Postsurgical nPN n = 15	*p* value
n with delayed GE^b^ (%)	27 (59%)	58 (48%)	0.232	7 (44%)	36 (43%)	0.978	14 (64%)	8 (40%)	0.125	4 (67%)	12 (80%)	0.517
n delayed 2 h GE (%)	11 (24%)	37 (31%)	0.379	4 (25%)	22 (27%)	0.900	6 (27%)	4 (20%)	0.580	0 (0%)	9 (60%)	0.026*
GES retention at 2 h	48 ± 19	50 ± 21	0.366	48 ± 15	51 ± 17	0.373	47 ± 23	39 ± 26	0.217	45 ± 12	62 ± 25	0.102
n delayed 4 h GE (%)	26 (57%)	54 (45%)	0.200	6 (38%)	33 (40%)	0.881	14 (64%)	9 (45%)	0.004*	4 (67%)	12 (80%)	0.371
GES retention at 4 h	17 ± 19	18 ± 21	0.471	13 ± 13	14 ± 16	0.937	19 ± 23	18 ± 24	0.325	18 ± 12	37 ± 29	0.102

Correlation values	Pearson's (All Patients)		Pearson's (Idiopathic)		Pearson's (Diabetic)		Pearson's (Postsurgical)					
Correlation between GES & PN at 4 h	0.02	0.801	− 0.03	0.768	0.281	0.072	0.372	0.096				
Correlation between GES & PN at 4 h	0.06	0.445	− 0.071	0.489	0.248	0.112	− 0.233	0.310				

^a^"All patients" include 9 diabetic and postsurgical patients whose group was too small for independent statistical analysis

^b^"Delayed GE" defined as being delayed at either 2 h (> 60%) or 4 h (> 10%)

Inclusive of patients with all etiologies, 27/85 (32%) had delayed emptying and PN, 58/85(68%) had delayed emptying but nPN, 19/81 (23%) had normal emptying and PN, and 62/81 (77%) had normal emptying but nPN (*p* = 0.232). There was a similar prevalence of delayed GE for those with PN and nPN among each etiological group except for diabetic patients. There were no significant correlations between the severity of PN and gastric retention at two nor four hours in any of the cohorts.

### Comparing symptoms (GCSI, PAGI-SYM, HADS, neuropathic) among PN vs. nPN

Table 2 compares the GCSI, PAGI-SYM, and HADS symptom scores between PN vs. nPN, inclusive of patients who did not undergo GES. All patients with PN reported on average a more severe GCSI total score (3.40 ± 1.02 in PN vs. 2.98 ± 0.97 in nPN, *p* < 0.005) and PAGI-SYM total score (3.12 ± 0.99 vs. 2.58 ± 0.93, *p* < 0.005) than their counterparts with nPN. Specific symptoms that were significantly worse in PN vs. nPN: vomiting (2.75 ± 1.82 vs. 2.19 ± 1.92, *p* = 0.035), bloating (3.63 ± 1.49 vs. 3.12 ± 1.56, *p* = 0.007), abdominal distension (3.37 ± 1.68 vs. 2.64 ± 1.67, *p* < 0.005), and upper abdominal pain (3.27 ± 1.55 vs. 2.70 ± 1.79, *p* = 0.029).

**Table 2:** Symptom severity of patients with symptoms of gastroparesis (PAGI-SYM, GCSI, HADS)

	All			Idiopathic			Diabetic			Post-Surgical		
	*n* = *250*			*n* = *148*			*n* = *61*			*n* = *32*		
	PN	nPN		PN	nPN		PN	nPN		PN	nPN	
	*n* = *70*	*n* = *180*	*p* value	*n* = *22*	*n* = *126*	*p* value	*n* = *33*	*n* = *28*	*p value*	*n* = *11*	*n* = *21*	*p value*
HADS Score- Anxiety^a^	8.28 ± 4.26	6.76 ± 4.38	0.007*	8.75 ± 4.77	6.65 ± 4.43	0.034*	8.74 ± 3.83	7.11 ± 4.16	*0.084*	6.91 ± 4.44	7.45 ± 4.39	*0.812*
*n* no case of anxiety (%)	31 (44%)	114 (63%)	0.006*	7 (32%)	80 (63%)	0.005*	14 (42%)	16 (57%)	*0.787*	7 (64%)	14 (67%)	*0.864*
*n* borderline/abnormal anxiety (%)	39 (56%)	66 (37%)		15 (68%)	46 (37%)		19 (58%)	12 (43%)		4 (36%)	7 (33%)	
HADS Score- Depression^a^	8.11 ± 4.26	6.27 ± 4.75	0.002*	6.82 ± 4.28	5.91 ± 4.42	0.305	8.67 ± 4.26	7.39 ± 4.65	*0.187*	8.77 ± 3.84	7.88 ± 6.43	*0.525*
*n* no case of depression (%)	32 (46%)	114 (63%)	0.011*	12 (55%)	81 (64%)	0.383	13 (39%)	17 (61%)	*0.097*	6 (55%)	11 (52%)	*0.907*
*n* borderline/abnormal depression (%)	38 (54%)	66 (37%)		10 (45%)	45 (36%)		20 (61%)	11 (39%)		5 (45%)	10 (48%)	
GCSI Score^b,d^	3.40 ± 1.02	2.98 ± 0.97	< 0.005*	3.70 ± 0.83	2.88 ± 0.98	< 0.005*	3.28 ± 1.07	3.17 ± 0.94	*0.452*	3.31 ± 1.32	3.23 ± 0.94	*0.475*
PAGI-SYM Total Score^c,d^	3.12 ± 0.99	2.58 ± 0.93	< 0.005*	3.50 ± 0.72	2.53 ± 0.94	< 0.005*	2.91 ± 1.06	2.70 ± 0.87	*0.167*	3.02 ± 1.26	2.74 ± 0.97	*0.275*
Gastrointestinal Symptom Details												
Nausea	3.67 ± 1.32	3.39 ± 1.44	0.170	3.95 ± 1.13	3.38 ± 1.37	0.057	3.65 ± 1.43	3.43 ± 1.45	*0.478*	3.32 ± 1.49	3.29 ± 1.85	*0.744*
Retching	2.60 ± 1.62	2.12 ± 1.76	0.052	2.95 ± 1.76	2.04 ± 1.71	0.020*	2.66 ± 1.54	2.25 ± 1.88	*0.481*	2.05 ± 1.52	2.00 ± 1.95	*0.984*
Vomiting	2.75 ± 1.82	2.19 ± 1.92	0.035*	2.76 ± 1.87	2.07 ± 1.91	0.105	2.94 ± 1.80	2.18 ± 2.00	*0.122*	2.59 ± 1.93	2.63 ± 1.91	*0.888*
Stomach Fullness	3.71 ± 1.44	3.57 ± 1.29	0.219	3.95 ± 1.29	3.43 ± 1.34	0.051	3.48 ± 1.56	3.57 ± 1.20	*0.791*	3.96 ± 1.55	4.24 ± 0.94	*0.862*
Feeling excessively full	3.98 ± 1.25	3.68 ± 1.43	0.107	4.14 ± 1.13	3.54 ± 1.55	0.089	3.86 ± 1.38	3.74 ± 1.17	*0.382*	4.09 ± 1.22	4.27 ± 0.85	*0.881*
Not able to finish a meal	3.69 ± 1.41	3.59 ± 1.49	0.692	3.77 ± 1.41	3.61 ± 1.49	0.682	3.41 ± 1.48	3.29 ± 1.46	*0.689*	4.27 ± 1.27	4.05 ± 1.20	*0.460*
Loss of appetite	3.52 ± 1.41	3.20 ± 1.63	0.215	3.77 ± 1.38	3.22 ± 1.60	0.618	3.42 ± 1.32	2.82 ± 1.66	*0.15*	3.64 ± 1.57	3.76 ± 1.64	*0.658*
Bloating	3.63 ± 1.49	3.12 ± 1.56	0.007*	4.05 ± 1.17	2.99 ± 1.61	0.002*	3.36 ± 1.64	3.57 ± 1.37	*0.892*	3.55 ± 1.69	3.40 ± 1.36	*0.541*
Abdominal distension	3.37 ± 1.68	2.64 ± 1.67	< 0.005*	3.86 ± 1.49	2.45 ± 1.68	< 0.005*	3.08 ± 1.70	3.50 ± 1.45	*0.372*	3.30 ± 1.69	2.52 ± 1.57	*0.154*
Upper abdominal pain	3.27 ± 1.55	2.70 ± 1.79	0.029*	3.64 ± 1.22	2.71 ± 1.78	0.030*	2.97 ± 1.74	2.96 ± 1.88	*0.900*	3.27 ± 1.68	2.48 ± 1.57	*0.147*
Upper abdominal discomfort	3.32 ± 1.50	2.97 ± 1.65	0.156	3.50 ± 1.37	3.00 ± 1.63	0.213	3.17 ± 1.61	3.04 ± 1.71	*0.830*	3.27 ± 1.68	2.81 ± 1.54	*0.362*
Lower abdominal pain	2.93 ± 1.60	1.84 ± 1.49	< 0.005*	3.75 ± 1.21	1.83 ± 1.44	< 0.005*	2.38 ± 1.61	1.93 ± 1.61	*0.279*	3.00 ± 1.79	1.95 ± 1.66	*0.107*
Lower abdominal discomfort	3.05 ± 1.56	1.98 ± 1.51	< 0.005*	3.75 ± 1.21	1.95 ± 1.45	< 0.005*	2.67 ± 1.61	2.04 ± 1.57	*0.119*	3.00 ± 1.79	2.35 ± 1.77	*0.315*
Heartburn during the day	2.06 ± 1.54	1.97 ± 1.63	0.684	2.09 ± 1.69	2.00 ± 1.58	0.866	2.00 ± 1.48	1.79 ± 1.69	*0.579*	2.18 ± 1.60	1.86 ± 1.71	*0.569*
Recumbent heartburn	1.94 ± 1.68	1.96 ± 1.70	0.987	1.82 ± 1.62	1.97 ± 1.63	0.680	1.94 ± 1.64	1.57 ± 1.81	*0.294*	2.00 ± 2.00	2.19 ± 1.75	*0.745*
Daytime chest discomfort	2.19 ± 1.50	1.51 ± 1.49	0.001*	2.45 ± 1.41	1.50 ± 1.50	0.008*	2.18 ± 1.55	1.32 ± 1.47	0.023*	1.64 ± 1.50	1.67 ± 1.28	*0.889*
Recumbent chest discomfort	2.09 ± 1.47	1.41 ± 1.56	< 0.005*	2.14 ± 1.28	1.41 ± 1.57	0.020*	2.06 ± 1.52	1.07 ± 1.51	0.007*	1.73 ± 1.68	1.71 ± 1.42	*0.919*
Daytime reflux	2.46 ± 1.72	2.09 ± 1.73	0.127	3.45 ± 1.44	2.13 ± 1.70	0.001*	1.94 ± 1.73	2.14 ± 1.60	*0.605*	1.82 ± 1.60	1.95 ± 1.83	*0.920*
Nighttime reflux	2.30 ± 1.71	2.10 ± 1.73	0.337	3.14 ± 1.67	2.08 ± 1.70	0.006*	1.76 ± 1.58	1.89 ± 1.64	*0.796*	2.09 ± 1.81	2.57 ± 1.94	*0.493*
Bitter taste	2.37 ± 1.63	1.96 ± 1.63	0.068	3.18 ± 1.37	1.93 ± 1.65	0.001*	2.12 ± 1.67	2.04 ± 1.43	*0.848*	1.91 ± 1.58	1.86 ± 1.62	*0.887*

^a^HADS scoring is based on 0–7 (no case), 8–10 (borderline abnormal), 11–21 (abnormal)

^b^GCSI includes nausea, retching, vomiting, stomach fullness, feeling excessively full, not able to finish a meal, loss of appetite, bloating, and abdominal distension

^c^PAGI-SYM includes these symptoms as well as upper/lower abdominal pain/discomfort, heartburn, chest discomfort, reflux, and bitter taste

^d^Scoring for GCSI, PAGI-SYM is based on numerical correlations: 0 = none, 1 = very mild, 2 = mild, 3 = moderate, 4 = severe, 5 = very severe

Among 148 patients with symptoms of IG, the following presented more severely in PN than nPN: nausea (3.95 ± 1.13 vs. 3.38 ± 1.37, *p* = 0.057), retching (2.95 ± 1.76 vs. 2.04 ± 1.71, *p* = 0.020), stomach fullness (3.95 ± 1.29 vs. 3.43 ± 1.34, *p* = 0.051), bloating (4.05 ± 1.17 vs. 2.99 ± 1.61, *p* = 0.002), abdominal distension (3.86 ± 1.49 vs. 2.45 ± 1.68, *p* < 0.005), upper abdominal pain (3.64 ± 1.22 vs. 2.71 ± 1.78, *p* = 0.030). It follows that GCSI (3.70 ± 0.83 vs. 2.88 ± 0.98, *p* < 0.005) and PAGI-SYM (3.50 ± 0.72 vs. 2.53 ± 0.94, *p* < 0.005) scores were also more severe in PN vs. nPN.

In patients with symptoms of DG, PAGI-SYM (2.91 ± 1.06 vs. 2.70 ± 0.87, *p* = 0.167), GCSI (3.28 ± 1.07 vs. 3.17 ± 0.94, *p* = 0.452), and individual symptoms scores were similar between PN and nPN. In patients with symptoms of PSG, PAGI-SYM (3.11 ± 1.28 vs. 2.68 ± 0.96, *p* = 0.275) and GCSI (3.43 ± 1.22 vs. 3.15 ± 0.95, *p* = 0.196) scores were similar, as were other GI symptoms.

Table 3 examines the 166 patients who underwent GES and first divides patients by delayed versus normal emptying, then within each group by PN or nPN. Among all patients who underwent GES, the GCSI score was more severe in both delayed GE (3.51 ± 0.86) and normal GE groups (3.42 ± 0.91) with PN than their counterparts with delayed GE and nPN (3.16 ± 0.97) and normal GE with nPN (2.90 ± 1.00, *p* = 0.034). Vomiting was especially more severe in patients with delayed GE and PN (*p* = 0.006). Nausea, retching, bloating, and abdominal distension tended to be higher among groups with PN than nPN, but stomach fullness and not being able to finish a meal were reported to have similar severity among all groups.

**Table 3:** Gastrointestinal symptom details of patients with symptoms of gastroparesis who underwent gastric emptying scintigraphy

	All (including DPSG)					Idiopathic				
	(n = 166)					(n = 99)				
	Delayed Emptying		Normal Emptying			Delayed Emptying		Normal Emptying		
	*n* = *85*		*n* = *81*			*n* = *43*		*n* = *56*		
	PN	nPN	PN	nPN	*p* value	PN	nPN	PN	nPN	*p* value
	(n = 27)	(n = 58)	(n = 19)	(n = 62)		(n = 7)	(n = 36)	(n = 9)	(n = 47)	
NTSS-6 score	11.40 ± 3.52	1.30 ± 1.94	9.50 ± 3.09	1.60 ± 2.01	< 0.005*	11.09 ± 2.05	1.36 ± 1.87	10.33 ± 3.28	1.42 ± 1.91	< 0.005*
Nausea	4.02 ± 1.06	3.45 ± 1.38	3.37 ± 1.34	3.28 ± 1.51	0.170	4.14 ± 1.21	3.58 ± 1.13	3.44 ± 1.24	3.18 ± 1.42	0.278
Retching	2.91 ± 1.72	2.45 ± 1.85	2.58 ± 1.43	1.90 ± 1.65	0.054	3.43 ± 2.37	2.56 ± 1.83	2.78 ± 1.48	1.83 ± 1.51	0.040*
Vomiting	3.31 ± 1.79	2.69 ± 1.84	2.42 ± 1.54	1.93 ± 1.86	0.006*	3.47 ± 1.87	2.53 ± 1.89	2.67 ± 1.80	1.87 ± 1.83	0.110
Stomach Fullness	3.78 ± 1.37	3.77 ± 1.12	3.79 ± 0.98	3.64 ± 1.12	0.802	4.29 ± 0.95	3.49 ± 1.20	3.67 ± 1.00	3.67 ± 1.02	0.402
Feeling excessively full	4.09 ± 1.24	3.77 ± 1.27	3.95 ± 1.13	3.65 ± 1.41	0.363	4.29 ± 1.11	3.53 ± 1.44	3.89 ± 1.27	3.63 ± 1.42	0.491
Not able to finish meal	3.59 ± 1.55	3.59 ± 1.41	3.82 ± 1.12	3.56 ± 1.48	0.979	3.29 ± 1.89	3.56 ± 1.50	3.78 ± 1.20	3.64 ± 1.37	0.983
Loss of appetite	3.67 ± 1.20	3.09 ± 1.57	3.79 ± 1.03	3.24 ± 1.71	0.316	3.14 ± 1.68	3.17 ± 1.50	4.11 ± 0.93	3.32 ± 1.64	0.410
Bloating	3.52 ± 1.50	3.32 ± 1.45	3.95 ± 1.13	3.11 ± 1.56	0.141	3.86 ± 1.77	3.26 ± 1.54	3.89 ± 0.78	3.02 ± 1.57	0.233
Abdominal distension	3.17 ± 1.75	2.88 ± 1.62	3.32 ± 1.67	2.47 ± 1.72	0.083	4.43 ± 0.53	2.89 ± 1.68	2.89 ± 1.90	2.26 ± 1.74	0.007*
Upper abdominal pain	3.41 ± 1.45	2.82 ± 1.83	3.26 ± 1.48	2.68 ± 1.78	0.318	4.14 ± 1.07	3.21 ± 1.65	3.33 ± 1.22	2.66 ± 1.80	0.118
Upper abd. discomfort	3.31 ± 1.48	2.86 ± 1.75	3.53 ± 1.12	3.02 ± 1.65	0.634	3.57 ± 1.90	3.37 ± 1.57	3.44 ± 1.01	2.91 ± 1.69	0.449
Lower abdominal pain	2.78 ± 1.67	1.81 ± 1.57	3.37 ± 1.22	1.95 ± 1.40	< 0.005*	3.14 ± 1.86	2.14 ± 1.57	4.06 ± 0.73	1.91 ± 1.38	< 0.005*
Lower abd. discomfort	2.89 ± 1.69	2.09 ± 1.62	3.61 ± 0.86	2.07 ± 1.40	< 0.005*	3.14 ± 1.86	2.31 ± 1.47	4.06 ± 0.73	2.10 ± 1.37	0.001*
Heartburn during the day	2.15 ± 1.61	2.33 ± 1.68	2.00 ± 1.80	1.80 ± 1.69	0.361	1.71 ± 1.70	2.58 ± 1.59	2.56 ± 2.07	1.84 ± 1.57	0.142
Recumbent heartburn	2.22 ± 1.72	2.38 ± 1.74	1.68 ± 1.80	1.65 ± 1.72	0.086	1.57 ± 1.72	2.53 ± 1.61	2.00 ± 2.00	1.68 ± 1.63	0.118
Daytime chest discomfort	2.30 ± 1.41	1.60 ± 1.45	2.21 ± 1.51	1.66 ± 1.65	0.115	2.43 ± 1.13	1.66 ± 1.45	2.67 ± 1.73	1.64 ± 1.65	0.215
Recumbent chest discomfort	2.30 ± 1.56	1.59 ± 1.67	2.11 ± 1.24	1.46 ± 1.63	0.042*	2.29 ± 1.25	1.72 ± 1.73	2.44 ± 1.42	1.47 ± 1.60	0.213
Daytime reflux	2.74 ± 1.77	2.58 ± 1.67	2.37 ± 1.83	2.04 ± 1.82	0.277	4.00 ± 0.82	2.85 ± 1.66	3.67 ± 1.66	2.14 ± 1.77	0.009*
Nighttime reflux	2.59 ± 1.80	2.74 ± 1.78	2.32 ± 1.80	1.77 ± 1.70	0.178	3.57 ± 1.62	2.83 ± 1.72	3.44 ± 1.59	1.94 ± 1.70	0.006*
Bitter taste	2.63 ± 1.62	2.13 ± 1.68	2.21 ± 1.75	1.66 ± 1.63	0.073	3.57 ± 0.98	2.23 ± 1.67	3.00 ± 1.66	1.62 ± 1.62	0.007*
GCSI Total Score	3.51 ± 0.86	3.16 ± 0.97	3.42 ± 0.91	2.90 ± 1.00	0.034*	3.86 ± 0.83	3.12 ± 1.05	3.40 ± 0.98	2.83 ± 0.98	0.065
PAGI-SYM Total Score	3.19 ± 0.93	2.73 ± 0.96	3.21 ± 0.79	2.54 ± 0.93	0.003*	3.55 ± 0.98	2.84 ± 1.01	3.41 ± 0.73	2.50 ± 0.93	0.010*

	Diabetic					Postsurgical				
	(n = 42)					(n = 21)				
	Delayed Emptying		Normal Emptying			Delayed Emptying		Normal Emptying		
	*n* = *22*		*n* = *20*			*n* = *16*		*n* = *5*		
	PN	nPN	PN	nPN	*p* value	PN	nPN	PN	nPN	*p* value
	(n = 14)	(n = 8)	(n = 8)	(n = 12)		(n = 4)	(n = 12)	(n = 2)	(n = 3)	
NTSS-6 score	11.68 ± 4.06	0.79 ± 1.53	9.24 ± 3.05	2.08 ± 2.27	< 0.005*	9.90 ± 3.24	0.86 ± 2.01	6.83 ± 0.24	2.44 ± 2.69	0.001*
Nausea	4.11 ± 0.96	3.50 ± 1.41	3.25 ± 1.67	3.17 ± 1.75	0.452	4.00 ± 1.15	2.83 ± 1.95	3.50 ± 0.71	5.00 ± 0.00	0.159
Retching	2.91 ± 1.46	3.00 ± 1.93	2.38 ± 1.60	1.67 ± 1.83	0.248	2.00 ± 1.63	1.50 ± 1.73	2.50 ± 0.71	3.33 ± 2.08	0.369
Vomiting	3.57 ± 1.74	2.88 ± 1.89	2.50 ± 1.20	1.92 ± 1.88	0.071	2.75 ± 2.22	2.86 ± 1.79	1.00 ± 1.41	2.67 ± 2.52	0.611
Stomach Fullness	3.50 ± 1.65	4.13 ± 0.64	3.63 ± 0.92	3.17 ± 1.34	0.377	4.25 ± 0.96	4.17 ± 0.94	5.00 ± 0.00	4.67 ± 0.58	0.553
Feeling excessively full	4.04 ± 1.42	3.88 ± 0.83	3.75 ± 1.04	3.50 ± 1.51	0.542	4.50 ± 0.58	4.06 ± 0.91	5.00 ± 0.00	4.67 ± 0.58	0.274
Not able to finish meal	3.29 ± 1.54	4.09 ± 0.66	3.58 ± 1.05	3.00 ± 1.86	0.819	4.75 ± 0.50	3.58 ± 1.38	5.00 ± 0.00	4.67 ± 0.58	0.15
Loss of appetite	3.71 ± 0.97	2.38 ± 1.41	3.25 ± 1.04	2.83 ± 1.95	0.170	4.25 ± 0.96	3.17 ± 1.90	4.50 ± 0.71	5.00 ± 0.00	0.273
Bloating	3.43 ± 1.50	3.63 ± 1.19	3.75 ± 1.49	3.33 ± 1.72	0.882	3.25 ± 1.71	3.17 ± 1.47	5.00 ± 0.00	4.33 ± 0.58	0.196
Abdominal distension	2.89 ± 1.92	3.75 ± 0.46	3.38 ± 1.41	3.42 ± 1.78	0.873	2.25 ± 2.06	2.00 ± 1.60	5.00 ± 0.00	2.67 ± 0.58	0.153
Upper abdominal pain	3.14 ± 1.66	2.75 ± 2.25	3.00 ± 1.85	2.50 ± 1.93	0.848	3.00 ± 1.41	1.83 ± 1.64	4.00 ± 1.41	3.67 ± 0.58	0.130
Upper abd. discomfort	3.39 ± 1.47	2.00 ± 2.00	3.25 ± 1.16	3.17 ± 1.64	0.338	2.75 ± 1.26	2.00 ± 1.41	5.00 ± 0.00	4.00 ± 1.00	0.031*
Lower abdominal pain	2.43 ± 1.70	1.25 ± 1.67	2.56 ± 1.12	2.00 ± 1.65	0.239	3.25 ± 1.71	1.33 ± 1.44	3.50 ± 2.12	2.33 ± 0.58	0.128
Lower abd. discomfort	2.64 ± 1.78	1.63 ± 1.85	3.13 ± 0.35	1.83 ± 1.64	0.166	3.25 ± 1.71	1.92 ± 1.93	3.50 ± 2.12	2.67 ± 0.58	0.435
Heartburn during the day	2.21 ± 1.63	2.50 ± 1.41	1.63 ± 1.60	1.33 ± 1.87	0.299	2.75 ± 2.06	1.08 ± 1.56	1.00 ± 0.00	4.00 ± 1.00	0.080
Recumbent heartburn	2.29 ± 1.73	2.25 ± 1.83	1.63 ± 1.60	1.25 ± 1.91	0.366	3.00 ± 2.16	1.58 ± 1.78	0.50 ± 0.71	4.00 ± 1.00	0.140
Daytime chest discomfort	2.50 ± 1.61	1.63 ± 1.41	1.88 ± 1.36	1.42 ± 1.78	0.313	1.25 ± 1.26	1.00 ± 1.13	1.50 ± 0.71	3.33 ± 0.58	0.076
Recumbent chest discomfort	2.43 ± 1.79	1.25 ± 1.39	1.88 ± 1.13	1.25 ± 1.91	0.194	3.00 ± 2.16	1.00 ± 1.35	1.50 ± 0.71	3.00 ± 1.00	0.169
Daytime reflux	2.07 ± 1.94	2.63 ± 1.41	1.38 ± 1.06	1.75 ± 1.91	0.488	2.75 ± 1.89	1.58 ± 1.73	0.50 ± 0.71	3.00 ± 2.65	0.490
Nighttime reflux	1.86 ± 1.70	2.88 ± 1.64	1.50 ± 1.41	1.25 ± 1.66	0.179	3.25 ± 2.22	2.08 ± 2.07	0.50 ± 0.71	2.67 ± 2.08	0.487
Bitter taste	2.43 ± 1.74	2.13 ± 1.36	1.75 ± 1.67	1.83 ± 1.70	0.730	2.50 ± 1.73	1.33 ± 1.50	0.50 ± 0.71	2.67 ± 2.08	0.253
GCSI Total Score	3.44 ± 0.92	3.48 ± 0.59	3.27 ± 0.94	2.92 ± 1.15	0.550	3.37 ± 0.91	2.91 ± 0.89	4.07 ± 0.06	3.97 ± 0.38	0.068
PAGI-SYM Total Score	3.06 ± 0.91	2.74 ± 0.88	2.91 ± 0.89	2.49 ± 1.00	0.366	3.11 ± 1.20	2.27 ± 0.80	3.51 ± 0.53	3.58 ± 0.24	0.028*

In patients with symptoms of IG, the GCSI score tended to follow a similar trend: patients with delayed GE and PN (3.86 ± 0.83) and patients with normal GE and PN (3.40 ± 0.98) reported more severe total scores than patients with delayed GE but nPN (3.12 ± 1.05) and normal GE but nPN (2.83 ± 0.98, *p* = 0.065). Retching and abdominal distension were especially more severe in patients with delayed GE and PN compared to with patients with normal GE and PN or any with nPN (*p* = 0.040, 0.007). In patients with symptoms of DG or PSG, GCSI and PAGI-SYM scores were more similar to each other among all subsymptoms.

Table 4 focuses on patients with delayed GE only. Among all patients with gastroparesis and delayed GE, GCSI and PAGI-SYM scores tended to be more severe among PN vs. nPN (3.51 ± 0.86 vs. 3.16 ± 0.97, *p* = 0.135 and 3.19 ± 0.93 vs. 2.73 ± 0.96, *p* = 0.024). Out of 43 patients with IG (symptoms of IG and delayed GE), seven (16%) patients reported PN and presented with more significant abdominal distension than their 36 (84%) nPN counterparts (4.43 ± 0.53 vs. 2.89 ± 1.68, *p* = 0.013). PAGI-SYM (3.55 ± 0.98 vs. 2.84 ± 1.01, *p* = 0.093) and GCSI (3.86 ± 0.83 vs. 3.12 ± 1.05, *p* = 0.073) scores tended to be more severe in IG-PN than IG-nPN but other symptoms were similar among this smaller sample size. Out of 22 patients with DG, 14 (64%) had PN and most symptoms were similar in severity compared to the eight (36%) with nPN. An exception was loss of appetite, which was more severe among DG-PN than DG-nPN (3.71 ± 0.97 vs. 2.38 ± 1.41, *p* = 0.020)*.*

**Table 4:** Gastrointestinal symptom details of patients who underwent gastric emptying scintigraphy with delayed emptying only

	All (including DPSG + PSG)			Idiopathic			Diabetic		
	Delayed Emptying			Delayed Emptying			Delayed Emptying		
	*n* = *85*			*n* = *43*			*n* = *22*		
	PN	nPN	*p value*	PN	nPN	*p value*	PN	nPN	*p value*
	*(n* = *27)*	*(n* = *58)*		*(n* = *7)*	*(n* = *36)*		*(n* = *14)*	*(n* = *8)*	
Nausea	4.02 ± 1.06	3.45 ± 1.38	0.076	4.14 ± 1.21	3.58 ± 1.13	0.2	4.11 ± 0.96	3.50 ± 1.41	0.336
Retching	2.91 ± 1.72	2.45 ± 1.85	0.286	3.43 ± 2.37	2.56 ± 1.83	0.133	2.91 ± 1.46	3.00 ± 1.93	0.626
Vomiting	3.31 ± 1.79	2.69 ± 1.84	0.111	3.47 ± 1.87	2.53 ± 1.89	0.14	3.57 ± 1.74	2.88 ± 1.89	0.245
Stomach Fullness	3.78 ± 1.37	3.77 ± 1.12	0.712	4.29 ± 0.95	3.49 ± 1.20	0.105	3.50 ± 1.65	4.13 ± 0.64	0.64
Feeling excessively full	4.09 ± 1.24	3.77 ± 1.27	0.122	4.29 ± 1.11	3.53 ± 1.44	0.14	4.04 ± 1.42	3.88 ± 0.83	0.384
Not able to finish a meal	3.59 ± 1.55	3.59 ± 1.41	0.826	3.29 ± 1.89	3.56 ± 1.50	0.825	3.29 ± 1.54	4.09 ± 0.66	0.481
Loss of appetite	3.67 ± 1.20	3.09 ± 1.57	0.119	3.14 ± 1.68	3.17 ± 1.50	0.96	3.71 ± 0.97	2.38 ± 1.41	0.020*
Bloating	3.52 ± 1.50	3.32 ± 1.45	0.409	3.86 ± 1.77	3.26 ± 1.54	0.206	3.43 ± 1.50	3.63 ± 1.19	0.883
Abdominal distension	3.17 ± 1.75	2.88 ± 1.62	0.278	4.43 ± 0.53	2.89 ± 1.68	0.013*	2.89 ± 1.92	3.75 ± 0.46	0.517
Upper abdominal pain	3.41 ± 1.45	2.82 ± 1.83	0.216	4.14 ± 1.07	3.21 ± 1.65	0.154	3.14 ± 1.66	2.75 ± 2.25	0.862
Upper abdominal discomfort	3.31 ± 1.48	2.86 ± 1.75	0.329	3.57 ± 1.90	3.37 ± 1.57	0.531	3.39 ± 1.47	2.00 ± 2.00	0.083
Lower abdominal pain	2.78 ± 1.67	1.81 ± 1.57	0.014*	3.14 ± 1.86	2.14 ± 1.57	0.139	2.43 ± 1.70	1.25 ± 1.67	0.102
Lower abdominal discomfort	2.89 ± 1.69	2.09 ± 1.62	0.041*	3.14 ± 1.86	2.31 ± 1.47	0.191	2.64 ± 1.78	1.63 ± 1.85	0.187
Heartburn during the day	2.15 ± 1.61	2.33 ± 1.68	0.555	1.71 ± 1.70	2.58 ± 1.59	0.155	2.21 ± 1.63	2.50 ± 1.41	0.555
Recumbent heartburn	2.22 ± 1.72	2.38 ± 1.74	0.656	1.57 ± 1.72	2.53 ± 1.61	0.125	2.29 ± 1.73	2.25 ± 1.83	0.972
Daytime chest discomfort	2.30 ± 1.41	1.60 ± 1.45	0.039*	2.43 ± 1.13	1.66 ± 1.45	0.172	2.50 ± 1.61	1.63 ± 1.41	0.217
Recumbent chest discomfort	2.30 ± 1.56	1.59 ± 1.67	0.042*	2.29 ± 1.25	1.72 ± 1.73	0.28	2.43 ± 1.79	1.25 ± 1.39	0.107
Daytime reflux	2.74 ± 1.77	2.58 ± 1.67	0.694	4.00 ± 0.82	2.85 ± 1.66	0.104	2.07 ± 1.94	2.63 ± 1.41	0.484
Nighttime reflux	2.59 ± 1.80	2.74 ± 1.78	0.739	3.57 ± 1.62	2.83 ± 1.72	0.225	1.86 ± 1.70	2.88 ± 1.64	0.187
Bitter taste	2.63 ± 1.62	2.13 ± 1.68	0.208	3.57 ± 0.98	2.23 ± 1.67	0.055	2.43 ± 1.74	2.13 ± 1.36	0.703
GCSI Score	3.51 ± 0.86	3.16 ± 0.97	0.135	3.86 ± 0.83	3.12 ± 1.05	0.073	3.44 ± 0.92	3.48 ± 0.59	0.891
PAGI-SYM Total Score	3.19 ± 0.93	2.73 ± 0.96	0.024*	3.55 ± 0.98	2.84 ± 1.01	0.093	3.06 ± 0.91	2.74 ± 0.88	0.195

Table 5 focuses on patients with normal GE only. Among all patients with symptoms of gastroparesis and normal GE, GCSI (3.42 ± 0.91 in PN vs. 2.90 ± 1.00 in nPN, *p* = 0.045) and PAGI-SYM (3.21 ± 0.79 vs. 2.54 ± 0.93, *p* = 0.006) scores were more severe in PN than nPN, and again, this pattern was reflected in the patients with symptoms of IG and normal emptying. However, this relationship was not apparent in the patients with symptoms of DG, in which all gastroparetic symptoms were similar in severity.

**Table 5:** Gastrointestinal symptom details of patients who underwent gastric emptying scintigraphy with normal emptying only

	All (including DPSG,PSG)			Idiopathic			Diabetic		
	*(n* = *166)*			*(n* = *99)*			*(n* = *42)*		
	Normal Emptying			Normal Emptying			Normal Emptying		
	*n* = *81*			*n* = *56*			*n* = *20*		
	PN	nPN	*p value*	PN	nPN	*p value*	PN	nPN	*p value*
	(n = 19)	(n = 62)		(n = 9)	(n = 47)		(n = 8)	(n = 12)	
Nausea	3.37 ± 1.34	3.28 ± 1.51	0.982	3.44 ± 1.24	3.18 ± 1.42	0.772	3.25 ± 1.67	3.17 ± 1.75	0.968
Retching	2.58 ± 1.43	1.90 ± 1.65	0.103	2.78 ± 1.48	1.83 ± 1.51	0.085	2.38 ± 1.60	1.67 ± 1.83	0.441
Vomiting	2.42 ± 1.54	1.93 ± 1.86	0.365	2.67 ± 1.80	1.87 ± 1.83	0.251	2.50 ± 1.20	1.92 ± 1.88	0.498
Stomach Fullness	3.79 ± 0.98	3.64 ± 1.12	0.734	3.67 ± 1.00	3.67 ± 1.02	0.879	3.63 ± 0.92	3.17 ± 1.34	0.551
Feeling excessively full	3.95 ± 1.13	3.65 ± 1.41	0.498	3.89 ± 1.27	3.63 ± 1.42	0.620	3.75 ± 1.04	3.50 ± 1.51	0.842
Not able to finish a meal	3.82 ± 1.12	3.56 ± 1.48	0.711	3.78 ± 1.20	3.64 ± 1.37	0.890	3.58 ± 1.05	3.00 ± 1.86	0.694
Loss of appetite	3.79 ± 1.03	3.24 ± 1.71	0.392	4.11 ± 0.93	3.32 ± 1.64	0.225	3.25 ± 1.04	2.83 ± 1.95	0.874
Bloating	3.95 ± 1.13	3.11 ± 1.56	0.029*	3.89 ± 0.78	3.02 ± 1.57	0.163	3.75 ± 1.49	3.33 ± 1.72	0.496
Abdominal distension	3.32 ± 1.67	2.47 ± 1.72	0.054	2.89 ± 1.90	2.26 ± 1.74	0.315	3.38 ± 1.41	3.42 ± 1.78	0.751
Upper abdominal pain	3.26 ± 1.48	2.68 ± 1.78	0.240	3.33 ± 1.22	2.66 ± 1.80	0.391	3.00 ± 1.85	2.50 ± 1.93	0.501
Upper abdominal discomfort	3.53 ± 1.12	3.02 ± 1.65	0.474	3.44 ± 1.01	2.91 ± 1.69	0.586	3.25 ± 1.16	3.17 ± 1.64	0.632
Lower abdominal pain	3.37 ± 1.22	1.95 ± 1.40	< 0.005*	4.06 ± 0.73	1.91 ± 1.38	< 0.005*	2.56 ± 1.12	2.00 ± 1.65	0.336
Lower abdominal discomfort	3.61 ± 0.86	2.07 ± 1.40	< 0.005*	4.06 ± 0.73	2.10 ± 1.37	< 0.005*	3.13 ± 0.35	1.83 ± 1.64	0.038*
Heartburn during the day	2.00 ± 1.80	1.80 ± 1.69	0.630	2.56 ± 2.07	1.84 ± 1.57	0.268	1.63 ± 1.60	1.33 ± 1.87	0.590
Recumbent heartburn	1.68 ± 1.80	1.65 ± 1.72	0.764	2.00 ± 2.00	1.68 ± 1.63	0.575	1.63 ± 1.60	1.25 ± 1.91	0.396
Daytime chest discomfort	2.21 ± 1.51	1.66 ± 1.65	0.157	2.67 ± 1.73	1.64 ± 1.65	0.104	1.88 ± 1.36	1.42 ± 1.78	0.341
Recumbent chest discomfort	2.11 ± 1.24	1.46 ± 1.63	0.053	2.44 ± 1.42	1.47 ± 1.60	0.090	1.88 ± 1.13	1.25 ± 1.91	0.137
Daytime reflux	2.37 ± 1.83	2.04 ± 1.82	0.438	3.67 ± 1.66	2.14 ± 1.77	0.019*	1.38 ± 1.06	1.75 ± 1.91	0.874
Nighttime reflux	2.32 ± 1.80	1.77 ± 1.70	0.208	3.44 ± 1.59	1.94 ± 1.70	0.016*	1.50 ± 1.41	1.25 ± 1.66	0.494
Bitter taste	2.21 ± 1.75	1.66 ± 1.63	0.230	3.00 ± 1.66	1.62 ± 1.62	0.029*	1.75 ± 1.67	1.83 ± 1.70	0.842
GCSI Score	3.42 ± 0.91	2.90 ± 1.00	0.045*	3.40 ± 0.98	2.83 ± 0.98	0.160	3.27 ± 0.94	2.92 ± 1.15	0.316
PAGI-SYM Total Score	3.21 ± 0.79	2.54 ± 0.93	0.006*	3.41 ± 0.73	2.50 ± 0.93	0.010*	2.91 ± 0.89	2.49 ± 1.00	0.217

All patients with PN reported more severe anxiety (8.28 ± 4.26) vs. nPN (6.76 ± 4.38, *p* = 0.007) with 39/70 (56%) PN presenting with a borderline or abnormal case of anxiety vs. 66/180 (37%) in nPN (*p* = 0.006) (Table 5). Depression was also more severe among PN (8.11 ± 4.26 vs. 6.27 ± 4.75, *p* = 0.002) with a similar distribution of 38/70 (54%) PN and 66/180 (37%) nPN presenting with borderline or abnormal cases (*p* = 0.011). Among patients with symptoms of IG, anxiety scores (8.75 ± 4.77 vs. 6.65 ± 4.43, *p* = 0.034) were more severe among patients with PN, 15/22 (68%) of whom presented with borderline or abnormal levels of anxiety compared to 46/126 (37%) nPN (*p* = 0.005), but depression scores were similar (6.82 ± 4.28 vs. 5.91 ± 4.42, *p* = 0.305). Anxiety and depression scores and distribution were similar in severity among diabetic and non-diabetic postsurgical subgroups.

Table 6 reports symptoms for the NTSS-6 in which total and individual scores of the NTSS-6 were similar in severity across all etiological groups (*p* = 0.989). There were no major differences among which specific symptoms of PN presented more often or more severely between groups.

**Table 6:** Details of neuropathic symptoms, as determined by the NTSS-6

	All	Idiopathic	Diabetic	Postsurgical	
	*n* = *70*	*n* = *22*	*n* = *33*	*n* = *11*	*p value*
TOTAL NTSS-6 SCORE	10.80 ± 3.92	10.57 ± 3.67	11.18 ± 4.31	10.20 ± 3.91	*0.989*
*Score details by symptom*					
1a. Frequency: deep aching, tightness, boring, pulling, or squeezing pain in feet/legs	1.43 ± 0.97	1.45 ± 0.86	1.48 ± 0.97	1.09 ± 1.14	0.695
1b. Severity of pain	1.90 ± 1.05	2.14 ± 0.89	1.97 ± 1.07	1.36 ± 1.21	0.351
Total Score #1	2.10 ± 1.20	2.32 ± 1.02	2.18 ± 1.23	1.51 ± 1.39	0.611
2a. Frequency: burning sensation in feet or legs	1.21 ± 1.08	1.14 ± 1.04	1.30 ± 1.13	0.91 ± 1.04	0.664
2b. Severity of burning	1.41 ± 1.06	1.36 ± 1.09	1.48 ± 1.03	1.09 ± 1.14	0.679
Total Score #2	1.56 ± 1.24	1.51 ± 1.27	1.62 ± 1.26	1.21 ± 1.28	0.582
3a. Frequency: "prickling" or "tingling" feeling in your feet or legs	1.50 ± 0.78	1.32 ± 0.84	1.48 ± 0.71	1.91 ± 0.83	0.435
3b. Severity of "prickling" or "tingling"	1.99 ± 0.86	2.00 ± 1.07	1.94 ± 0.83	2.09 ± 0.70	0.974
Total Score #3	2.18 ± 0.99	2.15 ± 1.20	2.13 ± 0.95	2.39 ± 0.88	0.909
4a. Frequency: asleep feeling, numbness, loss of sensation, but without "prickling"	1.11 ± 0.84	1.23 ± 0.92	0.97 ± 0.81	1.64 ± 0.50	0.028*
4b. Severity of numbness without "prickling"	1.67 ± 1.02	1.73 ± 1.03	1.63 ± 1.07	1.91 ± 0.83	0.741
Total Score #4	1.68 ± 1.22	1.83 ± 1.23	1.57 ± 1.25	2.12 ± 0.91	0.242
5a. Frequency: sharp, stabbing, shooting pain, or electric shock like pain	1.29 ± 0.85	1.05 0.95	1.45 ± 0.79	1.09 ± 0.83	0.260
5b. Severity of stabbing or electric like shock pain	1.81 ± 1.09	1.45 ± 1.18	2.03 ± 0.98	1.64 ± 1.21	0.290
Total Score #5	1.97 ± 1.21	1.57 ± 1.30	2.21 ± 1.11	1.76 ± 1.31	0.246
6a. Frequency: unusual sensitivity or tenderness when feet are touched or when walking	0.93 ± 0.86	0.77 ± 0.75	1.06 ± 0.90	0.91 ± 0.94	0.780
6b. Severity of sensitivity	1.23 ± 1.10	1.14 ± 1.04	1.38 ± 1.16	1.09 ± 1.14	0.907
Total Score #6	1.31 ± 1.22	1.18 ± 1.12	1.46 ± 1.31	1.21 ± 1.27	0.922

## Discussion

This study used the NTTS-6 questionnaire to assess for PN in patients with symptoms of gastroparesis. Our results suggest that PN is present not only in DG but is also present in IG, though less prevalent. Additionally, the cohort of patients with symptoms of IG and PN reported more severe manifestations of gastroparesis than patients with symptoms of IG but nPN.

In patients with symptoms of idiopathic gastroparesis, PN was present in 15% overall, with similar prevalence in patients with delayed GE (16%) and normal GE (16%). In patients with symptoms of diabetic gastroparesis, PN was present in 54% overall, being more frequent in patients with delayed GE (64%) than normal GE (40%). These numbers are slightly higher than a reported 45% prevalence of PN in a study focused on patients with symptoms of diabetic gastroparesis [9].

Our study found no correlation between peripheral neuropathy scores as assessed by the NTSS-6 and gastric retention in any etiological group. The NIH Gastroparesis Consortium found that in diabetic patients, the presence of peripheral neuropathy was associated with increased gastric retention at 2 h (63.4% vs. 54.2%, *p* = 0.04) and at 4 h (38.4% vs. 27.6%; *p* = 0.07) [9]. This contrast reflects an inconclusive trend in the literature regarding the relationship of symptom severity to gastric retention. While GES is a helpful, objective measure of gastric dysmotility, it generally does not provide information reflecting other functional causes of gastroparesis symptoms, such as fundic accommodation and impaired pyloric relaxation, and reflects a measurement in time that may change while a patient continues to experience gastroparetic symptoms [9, 19].

Patients with symptoms of IG and PN reported “moderate-severe” gastrointestinal symptoms of Gp, on average, while those with nPN averaged “mild-moderate” symptoms. Especially significantly worse symptoms included retching, bloating, abdominal distension, and upper abdominal pain. In idiopathic patients with delayed GE, mainly abdominal distension was more severe. A study focusing on patients with gastroparesis from the community at large (inclusive of academic centers) found that nausea, abdominal pain, and vomiting were important symptoms for treatment from the patient’s perspective. Along with early satiety, these symptoms were found to significantly impact the quality of life [20].

In addition to more severe gastrointestinal symptoms, patients with PN reported more severe psychosocial symptoms correlating with an increased number of borderline abnormal and abnormal levels of anxiety and depression as compared to patients without PN. Of all patients with PN, 54–56% presented with borderline to abnormal anxiety and depression compared to 37% of patients with nPN. A 2017 systematic review found that as the severity of gastrointestinal symptoms from Gp worsened, so did the severity of symptoms of anxiety and depression, which were associated with a decreased quality of life [21]. This known association of psychosocial symptoms with gastrointestinal symptoms exists as a possible confounder in our study, which observed an association between PN and both more severe gastrointestinal and psychosocial symptoms.

The distinction between symptom severity among patients with PN and nPN was most pronounced in patients with symptoms of IG and less pronounced in diabetic or postsurgical patients. Examining GCSI subscores revealed similar severity reported in both PN and nPN among diabetic patients with symptoms of gastroparesis. This data correlates with the NIDDK Gastroparesis Clinical Research Consortium findings that symptom severity as assessed by the GCSI and PAGI-SYM were similar among 153 diabetic patients with gastroparesis with and without peripheral neuropathy, as assessed by the NTSS-6 [9].

The strength of this study was determining if IG patients might have PN, which is known to be present in DG but is not described in IG. Limitations included that this study used the NTSS-6, which was designed as the screening tool for diabetic peripheral neuropathy, although patients with symptoms of IG or DG experienced similar symptoms of peripheral neuropathy that corresponded with the questionnaire. For all peripheral neuropathies, objective autonomic testing may be helpful in distinguishing autonomic neuropathy, however, this testing is not as easily accessible as the NTSS-6 and is not usually available to asymptomatic patients, which is why the NTSS-6 was ultimately preferred as a screening tool for PN [22]. The assessment of PN which relied on patients’ reports allowed for recall bias in filling out the NTSS-6 and other questionnaires.

While a sample size of 250 patients with symptoms of gastroparesis provided ample data for analysis, the final sample size of patients with official idiopathic gastroparesis confirmed by delayed GE on GES was 7, which was significantly smaller. This may have contributed to statistical bias in the analysis. GES studies were reported at the two- and four-hours timestamps but not one-hour, precluding identification of patients with rapid emptying. Additionally, this study was performed at a tertiary academic medical center, where patients tend to have more advanced symptoms and comorbidities than at other medical centers.

In this study, idiopathic patients with symptoms of gastroparesis and PN reported significantly more severe bloating, abdominal distension, and upper abdominal pain than idiopathic patients with nPN. This contrast allows for the idea that idiopathic patients with symptoms of gastroparesis can potentially benefit from neuromodulator pharmacotherapy, which are often used to treat painful peripheral neuropathy and are useful as ancillary therapies for symptoms of gastroparesis: nausea, vomiting, and abdominal pain. Pharmacotherapies such as gabapentin, pregabalin are prescribed for symptoms of abdominal pain, while mirtazapine is often used for symptoms of nausea and vomiting. Tricyclic antidepressants may modify nerve functions in the gut that depend on serotonin, which can blunt the effects of perceived pain or distension [23]. While a randomized trial of the tricyclic antidepressant nortriptyline in patients with IG did not demonstrate a significant improvement in overall symptoms of IG, there was a significant decrease in the severity of abdominal pain and early satiety [24]. Neuromodulators help alleviate symptoms in a related disorder, functional dyspepsia, where patients with more severe abdominal pain were more likely to report an improvement in symptoms if given amitriptyline than patients given a placebo [25]. Functional dyspepsia and gastroparesis are understood to be on a spectrum, and autonomic dysfunction, which can be a progression of PN, is still observed in patients with IG [19, 26].

In our study, 3–10% of patients in either groups with or without peripheral neuropathy reported neuromodulator use, which were lower values than reported in a 2015 review of symptomatic management of gastroparesis, where 16% of idiopathic patients with gastroparesis and approximately 28% of diabetic patients with gastroparesis were using neuromodulators [23]. This observation can be attributed to the fact that patients received this questionnaire at their intake appointments before they may have been prescribed neuromodulator therapy. Identification of PN in patients with IG may point to group of patients with autonomic dysfunction in which neuromodulation may be helpful.

## Conclusions

This study demonstrates that PN is prevalent in patients with symptoms of gastroparesis, not only in diabetic patients, but also patients with symptoms of idiopathic gastroparesis, a specific group in which those with PN present with more severe gastrointestinal symptoms than those without PN. This relationship is clear although no correlation between the severity of peripheral neuropathy and gastric retention among patients with symptoms of idiopathic gastroparesis was found. Discerning this relationship allows physicians to further understand the range of gastroparetic symptoms that a patient may experience, regardless of the etiology. As current treatment for gastroparesis is focused on the relief of symptoms, screening for PN may help identify a gastroparesis cohort who can benefit from neuromodulator use.

## Data Availability

The datasets used and/or analyzed during this study are available from the corresponding author on reasonable request.

## Abbreviations

GE GES: Gastric emptying, gastric emptying study
DG: Diabetic gastroparesis
PSG: Postsurgical gastroparesis
IG: Idiopathic gastroparesis
PN: Peripheral neuropathy

## Patient questionnaires

NTSS-6: Neuropathic Total Symptom Score-6
PAGI-SYM: Patient Assessment of Gastrointestinal Disorders- Symptoms Severity Index
GCSI: Gastroparesis Cardinal Symptom Index
HADS: Hospital Anxiety and Depression Score
